# Diseases of the Blood and Nutrition, and Infectious Diseases

**Published:** 1887-04

**Authors:** 


### Reviews.

**Diseases of the Blood and Nutrition, and Infectious Diseases**; being Vol. IV. of "A Handbook of Practical Medicines." By DR. HERMANN EICHHORST, and Vol. XII. of Wood's Library for 1886. Illustrated. New York: William Wood & Co.

